# Meta-Analysis of Microarray Expression Studies on Metformin in Cancer Cell Lines

**DOI:** 10.3390/ijms20133173

**Published:** 2019-06-28

**Authors:** Hans-Juergen Schulten, Sherin Bakhashab

**Affiliations:** 1 Center of Excellence in Genomic Medicine Research, Department of Medical Laboratory Technology, Faculty of Applied Medical Sciences, King Abdulaziz University, P.O. Box 80216, Jeddah 21589, Saudi Arabia; 2 Biochemistry Department, King Abdulaziz University, P.O. Box 80218, Jeddah 21589, Saudi Arabia; 3 Institute of Cellular Medicine, Newcastle University, Newcastle Upon Tyne NE2 4HH, UK

**Keywords:** metformin, meta-analysis, microarray expression studies, cancer cell lines, pleiotropic effects, pathway and network analysis

## Abstract

Several studies have demonstrated that metformin (MTF) acts with variable efficiency as an anticancer agent. The pleiotropic anticancer effects of MTF on cancer cells have not been fully explored yet. By interrogating the Gene Expression Omnibus (GEO) for microarray expression data, we identified eight eligible submissions, representing five different studies, that employed various conditions including different cell lines, MTF concentrations, treatment durations, and cellular components. A compilation of the data sets of 13 different conditions contained 443 repeatedly up- and 387 repeatedly down-regulated genes; the majority of these 830 differentially expressed genes (DEGs) were associated with higher MTF concentrations and longer MTF treatment. The most frequently upregulated genes include DNA damage inducible transcript 4 (*DDIT4*), chromodomain helicase DNA binding protein 2 (*CHD2*), endoplasmic reticulum to nucleus signaling 1 (*ERN1*), and growth differentiation factor 15 (*GDF15*). The most commonly downregulated genes include arrestin domain containing 4 (*ARRDC4*), and thioredoxin interacting protein (*TXNIP*). The most significantly (*p*-value < 0.05, Fisher’s exact test) overrepresented protein class was entitled, nucleic acid binding. Cholesterol biosynthesis and other metabolic pathways were specifically affected by downregulated pathway molecules. In addition, cell cycle pathways were significantly related to the data set. Generated networks were significantly related to, e.g., carbohydrate and lipid metabolism, cancer, cell cycle, and DNA replication, recombination, and repair. A second compilation comprised genes that were at least under one condition up- and in at least another condition down-regulated. Herein, the most frequently deregulated genes include nuclear paraspeckle assembly transcript 1 (*NEAT1*) and insulin induced gene 1 (*INSIG1*). The most significantly overrepresented protein classes in this compilation were entitled, nucleic acid binding, ubiquitin-protein ligase, and mRNA processing factor. In conclusion, this study provides a comprehensive list of deregulated genes and biofunctions related to in vitro MTF application and individual responses to different conditions. Biofunctions affected by MTF include, e.g., cholesterol synthesis and other metabolic pathways, cell cycle, and DNA replication, recombination, and repair. These findings can assist in defining the conditions in which MTF exerts additive or synergistic effects in cancer treatment.

## 1. Introduction

Metformin (MTF) is derived from the legume *Galega officinalis*, and is approved by the FDA for the treatment of Type 2 diabetes (T2D) [1,2]. One of the main effects of MTF in diabetic patients is to lower glucose levels by reducing hepatic glucose release, resulting in a secondary reduction of insulin levels [3,4]. Initial observations that MTF exerts anticancer properties in diabetic cancer patients were supported by an animal cancer model [5]. Several in vitro and in vivo studies followed, demonstrating, for the most part and to a varying degree, the anticancer effects of MTF [5]. The beneficial effect of MTF in cancer depends in part on the affected tumor site [6]. Meta-analyses indicate that MTF exerts anticancer activities notably in, e.g., pancreas, gastrointestinal and colorectal cancer [2]. In addition, MTF is apparently associated with reduced overall mortality of cancer patients, which is likely supported by beneficial effects of MTF as hyperglycemia reduction, weight loss and vascular protection [7,8]. As side effects of MTF are rare, the drug has gained considerations for broader clinical implications, especially for combinatorial drug applications in cancer treatment [9].

MTF exerts its primary main effects on the molecular level as an oxidative phosphorylation (OXPHOS) inhibitor by reversibly inhibiting NADH dehydrogenase (mitochondrial complex I) activity of the respiratory chain resulting in suppression of ATP production. This affects key energy and metabolic processes such as the tricarboxylic acid cycle, fatty acid β-oxidation, gluconeogenesis, and glycolysis [10]. The AMP-activated protein kinase (AMPK) is a cellular key energy sensor that is activated by increased AMP/ATP and/or ADP/ATP ratios [11]. MTF exerts its pleiotropic effects through AMPK-dependent and independent molecular mechanisms. A central AMPK-dependent mechanism is the inhibition of proliferation-promoting mTOR signaling. Affected cellular fate processes, which are implicated in directing MTF anticancer effects include cell cycle, cell growth, epithelial-to-mesenchymal transition (EMT), autophagy, and apoptosis [10,12]. However, MTF anticancer effects are variable, context specific and not fully explored yet, and a better molecular characterization of MTF effects is necessary to support its application in clinical practice.

The objective of this meta-analysis was to provide a comprehensive view of the genes and biological functions that are deregulated in response to MTF treatment. This should enlarge our knowledge of the molecular events associated with MTF treatment and shed light into the known pleiotropic MTF effects. As microarray expression data on in vivo MTF applications are virtually absent, we focused in our study on complementary in vitro experiments. Based on our initial observation that a considerable number of genes are expressed under different conditions in opposite directions when compared to the respective untreated conditions, we analyzed these genes separately. In addition, we compared shorter with longer MTF treatment conditions to provide a brief overview on the consecutive sequences of molecular events.

## 2. Results

### 2.1. Meta-Analysis on Microarray Expression Data Sets

The meta-analysis on microarray expression studies included data sets where MTF-treated cancer cells were compared to MTF-untreated ones. The following cancer cell lines were employed, SK-4 esophageal cancer cells (Gene Expression Omnibus (GEO) submission GSE16816), MCF-7 breast cancer cells (GSE36847, GSE69845), LoVo colon carcinoma cells (GSE67342), HepaRG liver carcinoma cells (GSE69844), Ishikawa endometrial adenocarcinoma cells (GSE69849), HepG2 liver carcinoma cells (GSE69850), and HL60, KG1a, MOLM14, and U937 acute myeloid lymphoid (AML) cells (GSE97346) (Table 1) [13,14,15,16,17]. The investigated studies were processed using HumanRef-6 v2.0 expression BeadChips (GSE16816), HG-U133 Plus 2.0 microarrays (GSE36847 and GSE67342), HG-U219 microarrays (GSE69844, GSE69845, GSE69849, and GSE69850), and HuGene-2.0 ST microarrays (GSE97346).

### 2.2. Genes Either Up- or Down-Regulated in MTF-Treated vs. MTF-Untreated Conditions

**Figure 1:**
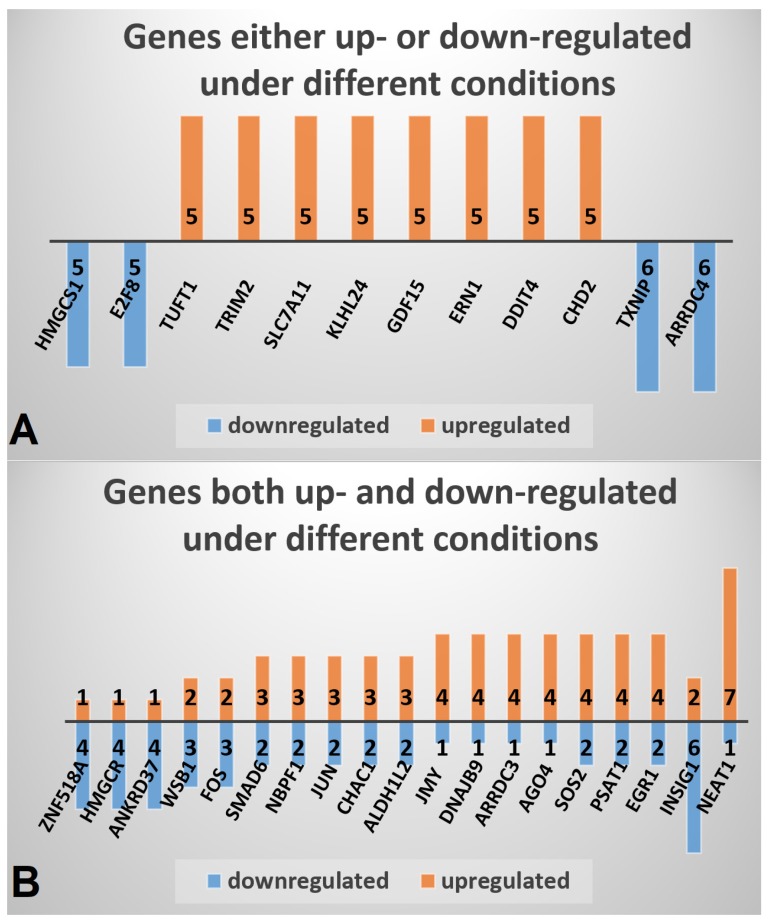
Genes most frequently differentially expressed under MTF treatment. The number of up- and/or down-regulated conditions is indicated at the frequency bars of each gene. (**A**) Genes either up- or down-regulated under different conditions. (**B**) Genes both up- and down-regulated under different conditions.

Using the data set of 13 different conditions (Table 1) a set of 830 significantly DEGs was compiled, comprising 443 up- and 387 down-regulated genes that were identified in MTF-treated compared to MTF-untreated cells in at least two different conditions (Table S1). The most frequently upregulated genes include chromodomain helicase DNA binding protein 2 (*CHD2*), DNA damage inducible transcript 4 (*DDIT4*), endoplasmic reticulum to nucleus signaling 1 (*ERN1*), growth differentiation factor 15 (*GDF15*), kelch like family member 24 (*KLHL24*), solute carrier family 7 member 11 (*SLC7A11*), tripartite motif containing 2 (*TRIM2*), and tuftelin 1 (*TUFT1*) (Figure 1A). The most frequently downregulated genes comprise arrestin domain containing 4 (*ARRDC4*), thioredoxin interacting protein (*TXNIP*), E2F transcription factor 8 (*E2F8*), and 3-hydroxy-3-methylglutaryl-CoA synthase 1 (*HMGCS1*).

### 2.3. Biofunctional Analysis on Genes Either Up- or Down-Regulated in MTF-Treated vs. MTF-Untreated Conditions

Interrogating the protein interaction database Biogrid with the set of 830 DEGs, 14 molecules, i.e., BAIAP2L1, CYCS, G3BP1, MARS, MCL1, MDM4, NOTCH1, PARP1, PPARGC1A, PPM1A, PRKAB1, SRPK2, TXNIP, and VCP were listed as AMPK interactors.

Individual biofunctional analysis of the 13 different conditions (Table 1) revealed that metabolic and cancer-related pathways were commonly listed among the top five pathways. Especially, different cholesterol biosynthesis pathways, the role of tissue factor in cancer, and unfolded protein response (URP) were listed each in three different conditions.

Taking the entire set of 830 DEGs, the protein class overrepresentation test indicated a 1.50-fold enrichment (FE) of nucleic acid binding proteins (*p*-value = 5.36 × 10^−4^), which were represented by 33 up- and 45 down-regulated factors. Canonical pathways significantly related to the upregulated factors include tRNA charging, whereas pathways related to the downregulated factors include cell cycle and DNA damage response mechanisms.

Top canonical pathways, associated with the entire 830 DEG set, comprised cholesterol biosynthesis and other metabolic pathways, as well as estrogen-mediated S phase entry (Table 2). Based on the expected vs. observed direction of pathway molecule expression, four of the five top pathways were all in a significant inhibition state. For example, the cholesterol pathway molecules in the data set included ACAT2, CYP51A1, DHCR7, DHCR24, HMGCS1, IDI1, LSS, MVD, MVK, and SQLE, which were all downregulated. Similarly, except the cell cycle inhibitor CDKN1A, all other molecules of the estrogen-mediated S phase entry pathway, which were present in the data set, i.e., CCNE1, CCNE2, E2F2, E2F3, E2F7, and E2F8 were downregulated.

**Figure 2:**
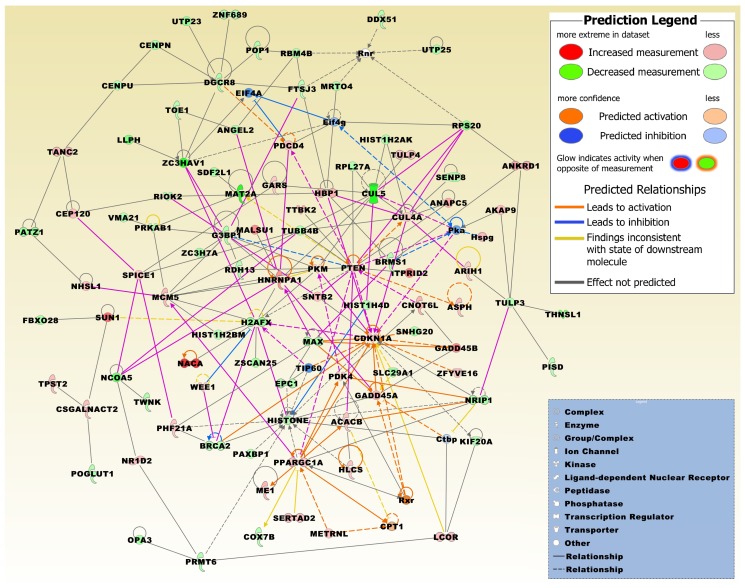
The merged network is based on the top three networks that were most significantly related to the microarray expression profiles of the 830 DEG set, which were either significantly up- or down-regulated in at least two different conditions. Network molecules are related to carbohydrate and lipid metabolism, small molecule biochemistry, cancer, cell cycle, cellular assembly and organization, DNA replication, recombination and repair, and molecular transport (Table 2). Upregulated molecules derived from the 830 DEG set include ACACB, AKAP9, ANAPC5, ANKRD1, ARIH1, ASPH, CDKN1A, CEP120, CNOT6L, CSGALNACT2, CUL4A, GADD45A, GADD45B, GARS, HBP1, HLCS, HNRNPA1, ITPRID2, LCOR, MALSU1, MCM5, ME1, METRNL, NACA, NHSL1, NR1D2, PDCD4, PDK4, PHF21A, PKM, PPARGC1A, PTEN, SERTAD2, SNTB2, SPICE1, SUN1, TANC2, TPST2, TTBK2, TULP4, WEE1, and ZFYVE16. Downregulated molecules include ANGEL2, BRCA2, BRMS1, CENPN, CENPU, COX7B, CUL5, DDX51, DGCR8, EPC1, FBXO28, FTSJ3, G3BP1, H2AFX, HIST1H2AK, HIST1H2BM, HIST1H4D, KIF20A, LLPH, MAT2A, MAX, MRTO4, NCOA5, NRIP1, OPA3, PATZ1, PAXBP1, PISD, POGLUT1, POP1, PRKAB1, PRMT6, RBM4B, RDH13, RIOK2, RPL27A, RPS20, SDF2L1, SENP8, SLC29A1, SNHG20, THNSL1, TOE1, TUBB4B, TULP3, TWNK, UTP23, UTP25, VMA21, ZC3H7A, ZC3HAV1, ZNF689, and ZSCAN25. The network was overlaid with the molecule activity predictor to calculate further molecular effects, as outlined in the prediction legend. Interconnecting factors added from the Ingenuity Knowledge Base include carnitine palmitoyltransferase 1A (CPT1), C-terminal-binding protein 1 (Ctbp), eukaryotic translation initiation factor 4A1 (EIF4A), eukaryotic translation initiation factor 4 gamma 1 (Eif4g), HISTONE, heparan sulfate proteoglycan (Hspg), Pka, ribosomal 45S RNA clusters (Rnr), retinoid receptor (Rxr), and Tat-interactive protein-60KDa (TIP60).

A merged network generated from the three top networks that were most significantly related to the DEG set displays significant relations with, e.g., carbohydrate and lipid metabolism, cancer, cell cycle, and DNA replication, recombination, and repair (Table 2, Figure 2).

The upstream regulators, which were most significantly related to the DEG set, were tosedostat (benzeneacetic acid), ATF4, TP53, GPER1, and SCAP (Figure S1). The first four upstream regulators were predicted to be in an activated state, whereas the last one was predicted to be in an inhibited state.

**Figure 3:**
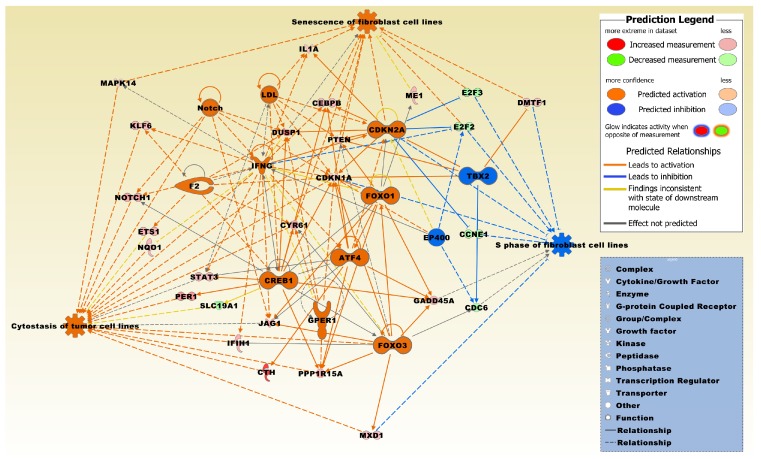
The regulator effects network had a consistency score of 7.3 and was significantly related to the microarray expression profiles of the 830 DEG set. Regulators include activating transcription factor 4 (ATF4), cyclin dependent kinase inhibitor 2A (CDKN2A), cAMP responsive element binding protein 1 (CREB1), E1A binding protein p400 (EP400), coagulation factor II, thrombin (F2), forkhead box O3 (FOXO3), G protein-coupled estrogen receptor 1 (GPER1), interferon gamma (IFNG), low density lipoprotein (LDL), notch receptor (Notch), and T-box 2 (TBX2). Molecules from the 830 DEG set involved in the network include CCNE1, CDC6, CDKN1A, CEBPB, CTH, CYR61, DMTF1, DUSP1, E2F2, E2F3, ETS1, FOXO1, GADD45A, IFIH1, IL1A, JAG1, KLF6, MAPK14, ME1, MXD1, NOTCH1, NQO1, PER1, PPP1R15A, PTEN, SLC19A1, and STAT3. The network was overlaid with the molecule activity predictor to calculate further molecular effects, as outlined in the prediction legend. The two functions, cytostasis of tumor cell lines and senescence of fibroblast cell lines, were predominantly activated whereas the function, S phase of fibroblast cell lines, was predominantly inhibited.

A regulator effects network was generated that interconnects regulator molecules significantly related to the DEG set with specific functions (Figure 3). Two of the three functions were predominately in an activated state and entitled, cytostasis of tumor cell lines, and senescence of fibroblast cell lines, whereas one function entitled, S phase of fibroblast cell lines, was predominately in an inhibited state.

### 2.4. Genes Both Up- and Down-Regulated in MTF-Treated vs. MTF-Untreated Conditions

To assess individual expression profiles in response to applied conditions, a second compilation was conducted containing a set of 411 DEGs that were at least under one condition up- and under at least one other condition down-regulated (Table S2). The most deregulated genes in this compilation include nuclear paraspeckle assembly transcript 1 (*NEAT1*), which was upregulated in seven conditions and downregulated in one, and insulin induced gene 1 (*INSIG1*), which was upregulated in two and downregulated in six conditions (Figure 1B). Other frequently deregulated genes include early growth response 1 (*EGR1*), phosphoserine aminotransferase 1 (*PSAT1*), and SOS Ras/Rho guanine nucleotide exchange factor 2 (*SOS2*).

### 2.5. Biofunctional Analysis of Genes Both Up- and Down-Regulated in MTF-Treated vs. MTF-Untreated Conditions

In the 411 DEG set, ubiquitin-protein ligases (*p*-value = 1.63 × 10^−4^; FE, 4.89), nucleic acid binding factors (*p*-value = 1.74 × 10^−4^; FE, 1.73), and mRNA processing factors (*p*-value = 2.04 × 10^−4^; FE, 3.91) represented the most significantly overrepresented protein classes. Top canonical pathways were significantly related to various signaling pathways including those involving prolactin, IL-8, HGF, PDGF, and ERBB signaling (Table 2).

**Figure 4:**
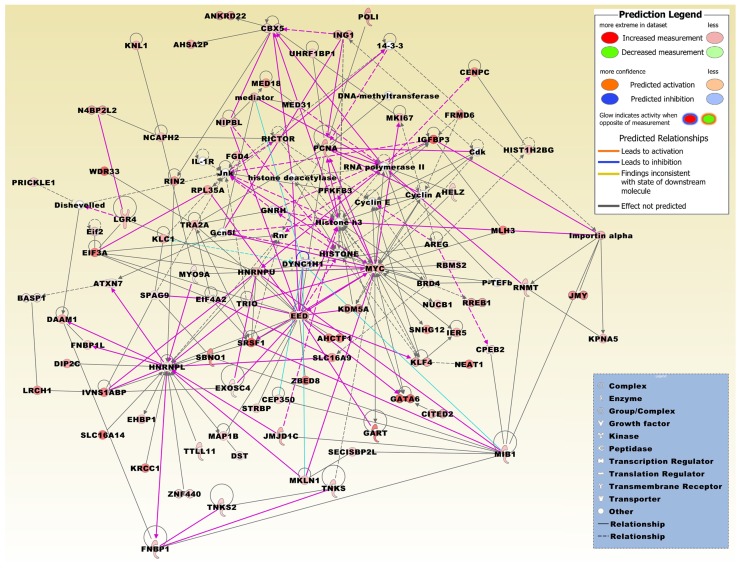
The merged network is based on the top three networks that were most significantly related to the microarray expression profiles of the set of 411 DEGs, which were both up- and down-regulated in at least two different conditions. Network molecules were related to, e.g., post-transcriptional and post-translational modification, DNA replication, recombination, and repair, cell-to-cell communication, cancer and other abnormalities, cellular growth, and carbohydrate metabolism (Table 2). Molecules derived from the 411 DEG set include AHCTF1, AHSA2P, ANKRD22, AREG, ATXN7, BASP1, BRD4, CBX5, CENPC, CEP350, CITED2, CPEB2, DAAM1, DIP2C, DST, DYNC1H1, EED, EHBP1, EIF3A, EIF4A2, EXOSC4, FGD4, FNBP1, FNBP1L, FRMD6, GART, GATA6, HELZ, HIST1H2BG, HNRNPL, HNRNPU, IER5, IGFBP3, ING1, IVNS1ABP, JMJD1C, JMY, KDM5A, KLC1, KLF4, KNL1, KPNA5, KRCC1, LGR4, LRCH1, MAP1B, MED18, MED31, MIB1, MKI67, MKLN1, MLH3, MYC, MYO9A, N4BP2L2, NCAPH2, NEAT1, NIPBL, NUCB1, PCNA, PFKFB3, POLI, PRICKLE1, RBMS2, RICTOR, RIN2, RNMT, RPL35A, RREB1, SBNO1, SECISBP2L, SLC16A14, SLC16A9, SNHG12, SPAG9, SRSF1, STRBP, TNKS, TNKS2, TRA2A, TRIO, TTLL11, UHRF1BP1, WDR33, ZBED8, and ZNF440. The network was overlaid with the molecule activity predictor to calculate further molecular effects, as outlined in the prediction legend. Interconnecting factors added from the Ingenuity Knowledge Base include 14-3-3 protein, cyclin dependent kinases (Cdk), cyclin A, cyclin E, dishevelled, DNA-methyltransferase, eukaryotic translation initiation factor 2 subunit alpha (Eif2), lysine acetyltransferase 2B (KAT2B; alias, Gcn5l), gonadotropin releasing hormone 1 (GNRH), HISTONE, histone deacetylase, histone h3, IL-1R, importin alpha, Jnk, mediator multiprotein complex, P-TEFb complex subunits, RNA polymerase II, and ribosomal 45S RNA clusters (Rnr).

A merged network based on the 411 DEG set and generated from the top three networks displays significant relations to diverse network functions, including cancer, post-transcriptional and post-translational modification, DNA replication, recombination, and repair, and carbohydrate metabolism (Table 2, Figure 4). Interrogating the Biogrid database with the DEG set, FOS and HNRNPL were listed as AMPK interactors.

### 2.6. DEGs in 6 h and 8 h vs. 24 h MTF Treatment

In a subset analysis, we compared the expression profiles in 6 h and 8 h vs. 24 h MTF treatment conditions. Only three DEGs were repeatedly observed in the shorter but not in the longer MTF treatment group. These three genes, which were coiled-coil domain containing 91 (*CCDC91*), growth differentiation factor 15 (*GDF15*), and pyruvate kinase M1/2 (*PKM*), were all upregulated. In contrast, more than 200 genes were differentially expressed in at least two 24 h MTF treatment conditions but in none of the 6 h and 8 h treatment conditions.

In the 24 h MTF treatment group, the most commonly deregulated genes include ASH1L antisense RNA 1 (*ASNS*), uncharacterized loci LOC101927372, phosphoserine aminotransferase 1 pseudogene 3 (*PSAT1P3*), RNA, U6 small nuclear 945, pseudogene (*RNU6-945P*), and UL16 binding protein 1 (*ULBP1*). The most significantly (*p* = 7.37 × 10^−5^; FE, 12.4) overrepresented protein class in this group of repeatedly deregulated molecules were histones, that were represented by five downregulated members, i.e., histone cluster 1 H4 family member d (HIST1H4D), histone cluster 1 H2B family member m (HIST1H2BM), H2A histone family member X (H2AFX), histone cluster 1 H2A family member b (HIST1H2AB), and histone cluster 1 H2A family member k (HIST1H2AK).

## 3. Discussion

Although several in vitro studies have performed expression analysis on selected genes under MTF-treated compared to MTF-untreated conditions, only a small subset of studies has applied mRNA expression profiling using whole transcriptome microarray technology. For example, one of the microarray expression studies, which is included in the present meta-analysis, demonstrated, by comparing the polysome-associated with the cytoplasmic RNA fraction, that the antiproliferative effect of MTF is mainly a result of translational suppression of mRNAs of cell cycle regulators and tumor promoters, including cyclin E2 (CCNE2) and ornithine decarboxylase 1 (ODC1), that are regulated via the mTORC1/eukaryotic translation initiation factor 4E-binding (4EBP) protein pathway [14]. In fact, *CCNE2* was significantly downregulated in our survey in four conditions. mTORC1 is known to enhance cell proliferation by inhibitory phosphorylation of 4EBP-1 and -2. In another microarray expression profiling study, the two hepatoma cell lines HepG2 and HepaRG were assessed for their capability of chemical hazard identification [18]. The results revealed remarkable differences of the two cell lines in response and discriminator capacity to carcinogen exposure, which might explain to some extent the differences in response to MTF treatment observed in our meta-analysis.

A part of the observed heterogeneous responses and pleiotropic effects related to MTF treatment may be attributed to the different applied in vitro methodologies and conditions [19]. For example, one of the assessed microarray expression studies applied 0.01 mM MTF for 6 h to four cell lines for the purpose to demonstrate expression signatures that most likely reflect direct molecular responses and mechanisms [16]. In our panel, all of these four cell lines displayed comparably lower numbers of DEGs.

### 3.1. Genes Upregulated under Different Conditions

DDIT4 (alias, REDD1) is known as a negative regulator of mTOR [20]. In MTF-treated LNCaP cells, DDIT4 inhibited mTOR, independently of AMPK, resulting in cell cycle arrest. ERN1 is a transmembrane and ER-stress regulated protein that is involved in the UPR and initial stage of autophagy [21]. A molecular genetic study in mouse embryonic stem cells demonstrated that functional Chd2 is necessary to mediate a chromatin structure that is associated with appropriate expression of developmentally regulated genes [22]. GDF15 has been described as a suitable serum biomarker for MTF usage in dysglycemic patients [23]. The ubiquitin ligase substrate receptor *KLHL24* has been identified as one of several overexpressed genes in a chemically induced hypoxia cell culture model [24]. The amino acid transporter SLC7A11 in expressed in various cancers, and supports cancer cells in detoxifying reactive oxygen species (ROS) [25]. Depending on its role in glucose and glutamine metabolism of cancer cells, the transporter can emerge as a target for anticancer treatment. In osteosarcoma, the E3-ubiquitin ligase TRIM2 is implicated in regulating development and metastasis, while its downregulation in clear cell renal cell carcinoma promoted cell proliferation, migration, and invasion and acted as an unfavorable prognostic indicator [26,27]. The acidic protein tuftelin 1 (TUFT1) is involved in mTORC1 activation by interacting with the RAB GTPase activating protein 1 (RABGAP1) and therefore may constitute a biomarker or a candidate for targeted therapy in mTOR activated, progressive cancers [28].

### 3.2. Genes Downregulated under Different Conditions

Both ARRDC4 and TXNIP are alpha-arrestin proteins known to regulate metabolic processes and are specifically involved in glucose uptake [29]. TXNIP is frequently downregulated in cancer by genetic and epigenetic mechanisms [30]. MTF can inhibit TXNIP expression, partly through AMPK [31]. Different members of the E2F transcription factors including *E2F8*, were repeatedly downregulated in the data sets of our survey. MTF treatment of lung cancer cells, in a similar manner as knockdown of *E2F8*, led to suppression of G1-S phase progression [32]. Microarray expression assays in rat FaO hepatoma cells demonstrated that MTF downregulates various metabolic genes and pathways including *Hmgcs1* and the cholesterol pathway by inhibiting nuclear receptor coactivator 2 (Ncoa2; alias, SRC-2) [33].

### 3.3. Genes Both Up- and Down-Regulated under Different Conditions

The noncoding RNA *Neat1* is a constituent of paraspeckles and induced by p53 in response to diverse stress signals, which support p53-mediated tumor suppression [34]. *INSIG1* encodes an endoplasmic reticulum (ER) membrane protein known to be involved in diverse metabolic processes. A microarray expression and qRT-PCR study has revealed that the gene is significantly downregulated under MTF treatment in triple-negative breast cancer cells and the glucose concentration may play a role in this process [35]. Zinc finger transcription factor EGR1 is known to be implicated in inflammatory processes, and is downregulated by AMPKα under hyperglycemic conditions [36]. EGR1 acts under certain conditions as a putative tumor suppressor [37]. *PSAT1* encodes a putative oncogene protein noted to be overexpressed and associated with unfavorable prognosis in a number of tumor types including breast and lung cancer [38,39]. SOS2 is a crucial factor for maintaining mitochondrial homeostasis [40]. Results of cell culture models assessing the transformation capacity of different RAS-mutant tumor cells indicated that SOS2 inhibition may emerge as a therapeutic option in *KRAS*-mutant cancers [41].

### 3.4. DEGs in 6 h and 8 h vs. 24 h MTF Treatment

CCDC91 is known as a clathrin adaptor accessory protein p56 that is involved in promoting membrane traffic through the trans-Golgi network; however, its function in cancer remains elusive [42]. As outlined earlier (Section 3.1), GDF15 is a suitable biomarker for MTF usage in glycemic patients. The molecule is a secreted ligand of the TGF-β superfamily. It exerts pleiotropic effects in various diseases and is rapidly induced as a stress response factor upon cellular injury and growth factor activity and is implicated in inflammatory and apoptotic pathways [43]. In T2D patients, increased circulating levels of GDF15 are associated with higher cancer incidence [44]. *PKM* expresses the two isoforms PKM1 and PKM2. PKM1 is constitutively active and promotes glucose catabolism, whereas PKM2 is activated only in response to increased levels of one or more allosteric activators [45]. The observed downregulation of five histone genes can be regarded as an epigenetic mechanism to modify gene expression pattern. Diverse epigenetic mechanisms including histone acetylation and methylation have been described as a result of MTF treatment [46].

### 3.5. Biofunctional Assessment

MTF is considered as a bioenergetic disruptor [47] and this meta-analysis is consistent with studies reporting that MTF targets several metabolic pathways; for example, a recent study reported that cholesterol biosynthesis pathway is affected by MTF treatment [33].

Biofunctional upstream analysis indicated that MTF and tosedostat exert overlapping metabolic effects. Tosedostat is known to inhibit a number of M1 aminopeptidase enzymes resulting in the depletion of amino acid pools, preferentially in cancer cells, which in consequence impairs cancer cell survival or proliferation.

Under glucose limitation in solid tumors, MTF inhibits the UPR, which is cytotoxic to cancer cells [48]. In fact, in our survey, a number of UPR-associated genes including *ERN1* and DNA damage inducible transcript 3 (*DDIT3*) were upregulated, or in case of *INSIG1*, up- and down-regulated under different conditions, although specific induction of the UPR by glucose deprivation was not an aim of the investigated microarray studies. Consistent with this and on basis of the 830 DEG set, the UPR pathway (*p* = 3.55 × 10^−4^) had neither a positive nor negative z-score.

Downregulation of the estrogen-mediated S phase entry in the 830 DEG set and deregulation of other, less significantly related, cell cycle pathways, including upregulation of the pathway entitled, cell cycle: G1/S checkpoint regulation (*p* = 2.45 × 10^−4^), is consistent with the observations that MTF affects cell cycle regulators in cancer cells [14,49].

### 3.6. Implications for MTF Treatment

One of the limitations of the meta-analysis concerns the analysis of only in vitro model systems that do not resemble entirely molecular mechanisms nor sequential events which are effective in complex biological environments such as cancer tissue. In particular, it has been criticized that suprapharmacological MTF concentrations, frequently used in cell culture experiments, exceed those observed in the in vivo environment, and therefore translation into clinical applications is limited [50]. Nonetheless, this meta-analysis provides an overview of the pleiotropic effects of MTF on the molecular level that in principle should also be operational in vivo. Based on the overrepresentation of nucleic acid binding factors in both major data sets, it can be contemplated that condition-specific pathways and networks are preferentially utilized on the level of gene regulation to route pleiotropic MTF effects.

Although many observational studies, especially in T2D patients and, to a lesser extent, clinical trials have reported that MTF is associated with reduced cancer risk, the major benefits of MTF emerge from its additive or synergetic effects in combinatorial anticancer therapies [19,51]. For example, application of MTF in MCF-7 cells with the hypoglycemia-mimicking compound 2-deoxy-D-glucose (2DG), which acts as a chemical UPR inducer, demonstrated that the drug increases the cytotoxic effects of 2DG in the breast cancer cells [52]. In this regard, a number of clinical cancer trials is assessing MTF in combination regimens [9]. To evaluate heterogenous responses to therapy, it can be envisaged to establish patient-derived cell culture models that may detect and explore molecular mechanisms or genomic biomarkers indicative for individual responses to clinical MTF applications [47,53].

## 4. Materials and Methods

### 4.1. Selection of Microarray Data Sets

Using the search string, metformin AND expression AND cancer AND human, in GEO [54] in November 2018, we retrieved eight submissions where MTF and control samples were employed as biological triplicates or higher number of replicates. Using the same search string in ArrayExpress [55] retrieved no additional data set.

Besides GEO submission GSE16816, all other submissions investigated more than one condition and each condition was analyzed separately, except in case of one study that used different MTF concentrations in its four submissions (GSE69844, GSE69845, GSE69849, and GSE69850). In these cases, we used the highest (0.01 mM) MTF condition for our meta-analysis. Conditions of other submissions comprised different exposure times to MTF, i.e., 8 h and 24 h (GSE67342), different cellular components, i.e., cytoplasmic and polysome-associated components (GSE36847), and different cell lines, i.e., HL60, KG1a, MOLM14, and U937 (GSE97346). In sum, 13 different conditions from eight GEO submission, representing five studies, were pooled and analyzed (Table 1). In a subset analysis, we compared DEGs of five conditions that used either 6 h or 8 h exposure time to MTF (GSE69844, GSE69845, GSE69849, GSE69850, and GSE67342) with five conditions that used 24 h exposure time to MTF (GSE67342 and GSE97346). In general, the meta-analysis adhered to recommendations outlined in a practical guideline for meta-analysis of gene expression microarray data sets [56].

### 4.2. Calculation of DEGs

For one submission (GSE16816), the fold change (FC) of DEGs was calculated between MTF-treated and MTF-untreated samples using the provided normalized intensity data. For all other submissions, the binary CEL files containing the intensity calculations based on the pixel values were imported into the Transcriptome Analysis Console (TAC) version 4.0.1 (Thermo Fisher Scientific, Waltham, MA, USA) that includes the LIMMA (linear modeling for microarrays) package from Bioconductor [57]. Based on the chosen parameters, the binary CEL files were normalized in TAC and lists of differentially expressed probe sets generated. A FC ≥ 1.5 and a *p*-value < 0.05 served as a threshold for statistical significance of expression data. A FC of ≥ 1.5 has been employed as a robust threshold for assessing MTF effects in in vitro model systems [58]. Where necessary, the BioMart community portal, Ensembl release 95, and the DAVID bioinformatics resources 6.8 were employed to convert microarray probe set IDs or alias symbols to official gene symbols [59,60,61]. Genes with both significantly up- and down-regulated probe sets in the same condition were excluded from further analysis.

### 4.3. Functional Gene Analysis

Pathway and network analyses were accomplished by using the Ingenuity Pathway Analysis (IPA) software (Qiagen, Hilden, Germany). IPA employs the curated Ingenuity Knowledge Base as a reference data set. The analysis settings included direct and indirect molecular relationships. Fisher’s exact test *p*-values indicated significant correlations between the analyzed data set molecules and functional frameworks prebuilt or generated de novo by IPA. Expression effects/coherence of expression effects of a molecule on other pathway or network molecules were predicted by using the molecule activity predictor. The canonical pathway workflow was utilized to determine those uploaded data set molecules that are co-expressed in a directional pathway. A z-score ≥ 2 predicts a significant activation state and ≤ −2 a significant inhibition state between expected and observed functional relationships. The number of data set molecules in relation to the entire set of pathway molecules is expressed as an overlap percentage. Network analysis was employed to explore significance of fit, expressed as a score value, between uploaded data set molecules and networks related to specific functions or diseases. Upstream analysis was utilized to interpret, by using z-scores, how differences in target gene expression are affected by upstream regulators. Regulator effects analysis was employed to interpret which regulators target uploaded data set molecules and which kind of downstream effects, i.e., diseases and/or functions, are associated. The extent to which a generated network is consistent with the Ingenuity Knowledge Base, i.e., either activated or inactivated, is expressed by a consistency score. The gene ontology (GO) online program PANTHER v. 11, which combines GO annotations and a phylogenetic tree model for inferring gene functions, was utilized to identify overrepresented protein classes in the data sets using Fisher’s exact test with a *p*-value < 0.05 for indicating statistical significance [62]. Biogrid, the database of physical and genetic interactions, was interrogated to identify AMPK interactors [63].

## Figures and Tables

**Table 1:** Microarray studies on MTF in vitro application included in the meta-analysis.

GEO ^1^ Data Set	Cell Lines	Treatment [h]	MTF [mM]	No. of DEGs	Array Platform	Date	Study
GSE16816	SK-4	12	5	101–500	HumanRef-6 v2.0 expression BeadChips	2011	[13]
GSE36847	MCF-7, cytoplasmic	12	10	501–1000	HG-U133 Plus 2.0	2012	[14]
GSE36847	MCF-7, polysome-associated	12	10	1001–3000	HG-U133 Plus 2.0	2012	[14]
GSE67342	LoVo	8	10	100–500	HG-U133 Plus 2.0	2015	[15]
GSE67342	LoVo	24	10	1001–3000	HG-U133 Plus 2.0	2015	[15]
GSE69844	HepaRG	6	0.01	<100	HG-U219	2016	[16]
GSE69845	MCF-7	6	0.01	<100	HG-U219	2016	[16]
GSE69849	Ishikawa	6	0.01	101–500	HG-U219	2016	[16]
GSE69850	HepG2	6	0.01	101–500	HG-U219	2016	[16]
GSE97346	HL60	24	10	1001–3000	HuGene-2.0 ST	2017	[17]
GSE97346	KG1a	24	10	101–500	HuGene-2.0 ST	2017	[17]
GSE97346	MOLM14	24	10	101–500	HuGene-2.0 ST	2017	[17]
GSE97346	U937	24	10	501–1000	HuGene-2.0 ST	2017	[17]

**^1^** Gene Expression Omnibus.

**Table 2:** Top pathways and networks in MTF-treated vs. MTF-untreated conditions.

Category	Genes Either Up- or Down-Regulated			Genes Both Up- and Down-Regulated		
	*p*-Value	Overlap [%]	z-Score/Score	*p*-Value	Overlap [%]	Score
**Top canonical pathways**						
Superpathway of cholesterol biosynthesis	7.29 × 10^−9^	35.7	−3.16			
Estrogen-mediated S phase entry	1.19 × 10^−5^	26.9	−2.65			
Oleate biosynthesis II (animals)	3.23 × 10^−5^	38.5	−1.34			
Cholesterol biosynthesis I	3.23 × 10^−5^	38.5	−2.24			
Mevalonate pathway I	3.23 × 10^−5^	38.5	−2.24			
Prolactin signaling				5.55 × 10^−9^	14.3	
IL-8 signaling				1.78 × 10^−8^	8.8	
HGF signaling				2.32 × 10^−8^	11.6	
PDGF signaling				1.25 × 10^−7^	12.2	
ERBB signaling				2.99 × 10^−7^	11.3	
**Top networks related to diseases and functions**						
Carbohydrate metabolism, small molecule biochemistry, cancer			58			
Cell cycle, cellular assembly and organization, DNA replication, recombination, and repair			44			
Cell cycle, lipid metabolism, molecular transport			44			
RNA post-transcriptional modification, cell cycle, DNA replication, recombination, and repair			42			
Hereditary disorder, neurological disease, organismal injury and abnormalities			42			
RNA post-transcriptional modification, DNA replication, recombination, and repair, cell-to-cell signaling and interaction						55
Cancer, gastrointestinal disease, organismal injury and abnormalities						47
Cellular growth and proliferation, post- translational modification, carbohydrate metabolism						44
RNA post-transcriptional modification, glomerular injury, organismal injury and abnormalities						40
Cancer, cell cycle, organismal injury and abnormalities						33

## Supplementary Materials

Supplementary Materials can be found at https://www.mdpi.com/1422-0067/20/13/3173/s1.

## Abbreviations

AMPK	AMP-activated protein kinase
DEG	differentially expressed gene
EMT	epithelial-to-mesenchymal transition
ER	endoplasmic reticulum
FC	fold change
FE	fold enrichment
GEO	Gene Expression Omnibus
GO	gene ontology
MTF	metformin
OXPHOS	oxidative phosphorylation
T2D	type 2 diabetes
UPR	unfolded protein response
